# Pre-Diagnosis Aspirin Use Has No Effect on Overall Survival in Patients With Colorectal Cancer: A Study of a Multi-Racial Population

**DOI:** 10.7759/cureus.22769

**Published:** 2022-03-02

**Authors:** Adham E Obeidat, Ratib Mahfouz, Gabriel Monti, Mahmoud M Mansour, Mohammad Darweesh, Jared Acoba

**Affiliations:** 1 Internal Medicine, University of Hawaiʻi, Honolulu, USA; 2 Internal Medicine, Kent Hospital, Warwick, USA; 3 Internal Medicine, Oregon Health & Science University, Portland, USA; 4 Internal Medicine, University of Missouri School of Medicine, Columbia, USA; 5 Internal Medicine, East Tennessee State University, Johnson City, USA; 6 Hematology and Oncology, The Queen's Medical Center, Honolulu, USA

**Keywords:** pd-1 inhibitors, nsaid, mortality, overall survival, colorectal cancer, aspirin

## Abstract

Introduction

Aspirin has been associated with a reduction in mortality in patients diagnosed with colorectal cancer (CRC). A possible mechanism for this is related to the programmed cell death 1 (PD-1) immune checkpoint pathway. Aspirin may have a synergistic effect with PD-1 inhibitors via inhibition of prostaglandin E2 (PGE2) production, which can reverse the ability of tumor cells to evade the immune system. This appears to be strongest in cancers that express PI3 kinase (PI3K) signaling activity, which aspirin downregulates. However, the benefit of pre-diagnosis aspirin use on CRC overall survival (OS) and cancer-specific survival is still controversial, and most studies have been performed in racially homogenous populations. Our study examines the effect of pre-diagnosis aspirin therapy on OS in a racially diverse group of patients with CRC.

Methods

This is a retrospective chart review of 782 patients diagnosed with CRC from January 2007 to December 2020. Kaplan-Meier curve was created to study the association of aspirin exposure compared to no exposure on OS. In addition, univariate and multivariate binary logistic regression analyses were done to investigate potential predictors of survival.

Results

Of the 782 patients with CRC, 55.1% were males, 22.2% whites, 58.5% Asians, and 17.7% Pacific-Islanders. Moreover, 38.4% of the patients had a history of aspirin use, 79% of them used it for more than one year. There were more patients with hypertension (HTN), hyperlipidemia (HLD), diabetes mellitus (DM), and chronic kidney disease (CKD) among those with a history of aspirin use. There was no difference in one, three, and five-year OS among aspirin users compared to non-users, p-value = 0.63. Age, grade, and stage were potential predictors of worsened OS. However, treatment with chemotherapy and CKD were potential predictors of worsened OS on univariate analysis only. No significant association was noticed with gender, tumor location, or other associated comorbidities.

Conclusion

The effect of pre-diagnosis aspirin use on CRC survival is not clear. In this retrospective analysis of a racially diverse population of CRC patients, we found that aspirin use was not associated with improved OS. Therefore, physicians should be careful about using aspirin as adjuvant therapy in CRC patients until high-quality prospective data are available, given the potential associated complications.

## Introduction

In 2021, the American Cancer Society estimated that there would be over 104,000 new cases of colon cancer and over 45,000 cases of rectal cancer [1]. Colorectal cancer (CRC) is the second leading cause of cancer death, accounting for nearly 10% of all cancer deaths, with an estimated total of over 52,000 deaths in the United States in 2021 [1]. As a result, there has been considerable investigation related to the screening and prevention of CRC. One agent that has been strongly linked to a possible reduction in the incidence of CRC is aspirin, an irreversible cyclooxygenase (COX) inhibitor that is among the most used drugs worldwide [2].

Aspirin has long been used for its antiplatelet effects in patients at increased risk for cardiovascular events. However, many studies have shown a potential benefit in preventing CRC [3-5]. Aspirin was the first pharmacological agent endorsed by the US Preventive Services Task Force for CRC screening for chemoprevention, with a 40% CRC risk reduction in individuals at average risk. However, data regarding this benefit are inconsistent [6]. It has also been suggested that post-diagnosis aspirin use can reduce mortality in patients with CRC [2]. A possible mechanism for this is related to the programmed cell death 1 (PD-1) immune checkpoint pathway. In addition, aspirin can reverse immune evasion of tumor cells through inhibition of prostaglandin E2 (PGE2) production [7]. There is growing evidence of possible synergism of PD-1 inhibitors and inhibition of PGE2 production via aspirin for possible combined immunotherapy strategies [7]. The post-diagnosis aspirin effect on CRC survival appears to be strongest in cancers that express PI3 kinase (PI3K) signaling activity [8]. However, the benefit of pre-diagnosis aspirin use on CRC overall survival (OS) and cancer-specific survival is still controversial [9]. In this study, we aim to examine the effect of pre-diagnosis aspirin use on OS in a racially diverse population of CRC patients.

This case was submitted as an abstract at the American College of Gastroenterology Annual Scientific Meeting, Las Vegas/Nevada, October 22-27, 2021.

## Materials and methods

Patients and data collection

We performed a retrospective analysis on data gathered from the Queen’s Medical Center (QMC), Honolulu, Hawaii, Oncology Data Registry (ODR). ODR was established in 1960 as part of the Hawaii Tumor Registry and has been contributing data to the Surveillance, Epidemiology, and End Results (SEER) program since 1973. All patients diagnosed with colorectal adenocarcinoma between January 1, 2007, and December 30, 2020, were eligible.

QMC Institutional Review Board (IRB) approval was obtained for conducting this study. Data on patient demographics (age, gender, race), clinicopathologic characteristics (stage, grade, site of the tumor, history of chemotherapy, history of surgery, and associated comorbidities such as hypertension [HTN], hyperlipidemia [HLD], diabetes mellitus [DM] and chronic kidney disease [CKD]), and survival were collected from ODR and medical records. In addition, data on aspirin use were collected via chart review to determine if patients were using aspirin at the time of their CRC diagnosis. The duration of aspirin use prior to diagnosis was also recorded. Finally, the race was self-reported by the patient. For the analysis, patients were categorized into three racial groups: Asian (Korean, Chinese, Japanese, Filipino, Asian Indian (Indian and Pakistani), Southeast Asian (Thai, Vietnamese, Cambodian, and Laotian), and other Asian), Pacific-Islander (Native Hawaiian, Samoan, Tongan, Micronesian, Marshallese, Fijian, Chamorro, and other Pacific-Islander), and White. Patients of other races or unknown races only made up 1.2% of our study population and were excluded from the analysis.

Statistics

The primary objective is to study the potential effects of aspirin use on OS among patients diagnosed with CRC. Descriptive statistics were used to evaluate characteristics of standard demographic, clinical, and tumor data. A two-sided p < 0.05 was considered statistically significant. OS was calculated using the Kaplan-Meier method, and univariate comparisons between groups were carried out using the log-rank test. Binary logistic regression models for survival were built to obtain OR and 95% CI adjusting for age, gender, race, histologic grade, stage, surgery, chemotherapy, aspirin use, and associated comorbidities. Statistical analyses and survival graphics were performed with SPSS version 25.0 (IBM Corp, Armonk, NY).

## Results

Demographic and clinical characteristics

A cohort of 1050 patients with CRC was identified, and 782 patients were included after excluding patients with missing data (n = 268). Table 1 shows the patients’ demographic and clinical characteristics. There were 431 males (55.1%). The median patients’ age was 67 (range: 27-100). Most of the patients were Asians (58.5%), while Whites constituted 22.2% and Pacific-Islanders 17.7%. Moreover, 694 patients (88.7%) had surgery, and 437 (55.8%) received chemotherapy. We identified 301 patients (38.4%) who were using aspirin at the time of diagnosis; 238 (79%) of them took aspirin for more than one year prior to diagnosis.

There was no significant difference in race, tumor location, grade, and stage among patients with a history of aspirin use compared to non-users. However, more patients with HTN, HLD, DM, and CKD were among those with a history of aspirin use.

**Table 1:** Demographic and clinical characteristics of patients with a history of aspirin use compared to non-users. Aspirin N: No history of aspirin use; Aspirin Y: History of aspirin use. N (%) = Number (Percentage) HTN: Hypertension; DM: Diabetes mellitus; HLD: Hyperlipidemia; CKD: Chronic kidney disease.

	Aspirin N N (%)	Aspirin Y N (%)
Age (p = 0.101)		
<65	240 (49.9%)	104 (34.55%)
>65	241 (50.1%)	197 (65.45%)
Gender (p = 0.756)		
Males	263 (54.68%)	168 (55.81%)
Females	218 (45.32%)	133 (44.19%)
Race (p = 0.955)		
White	107 (22.25%)	67 (22.26%)
Pacific	83 (17.26%)	56 (18.6%)
Asians	284 (59.04%)	174 (57.81%)
Others	7 (1.46%)	4 (1.33%)
Vital status (Dead) (p = 0.568)	237 (49.27%)	142 (47.18%)
Tumor grade (p = 0.085)		
Low (grade I & II)	368 (76.51%)	242 (80.40%)
High (grade III & IV)	113 (23.49%)	59 (19.6%)
Stage (p = 0.565)		
Early (stage I & II)	232 (48.23%)	151 (50.17%)
Late (stage III & IV)	249 (51.77%)	150 (49.83%)
Site (p = 0.498)		
Proximal	164 (34.1%)	119 (39.5%)
Distal	316 (65.8%)	182 (60.4%)
Chemotherapy (yes) (p = 0.131)	279 (58%)	158 (52.49%)
Surgery (yes) (p = 0.621)	429 (89.19%)	265 (88.04%)
HTN	236 (49.06%)	250 (83.06%)
DM	86 (17.88%)	123 (40.86%)
HLD	202 (42%)	232 (77.08%)
CKD	28 (5.82%)	69 (22.92%)

Overall survival in patients with aspirin users compared to non-users

**Figure 1:**
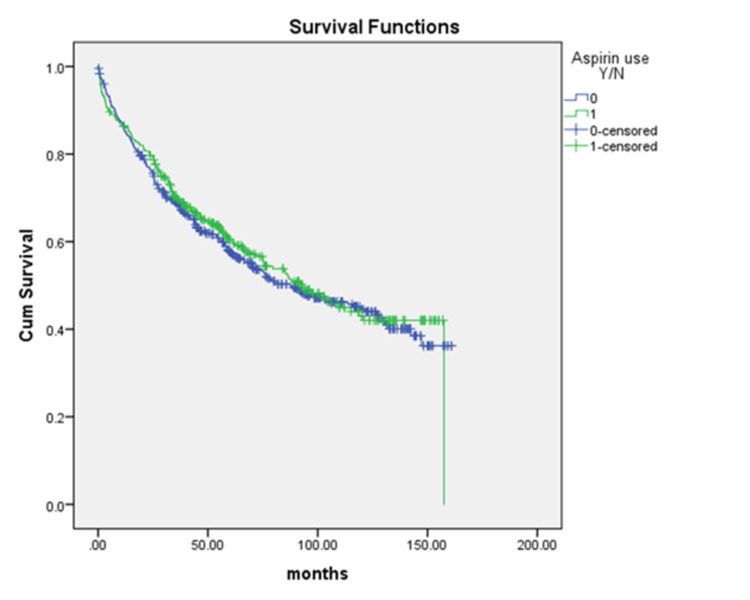
Kaplan-Meier survival curve for CRC patients with aspirin exposure vs. no exposure. CRC: Colorectal cancer.

We divided the patients according to aspirin use into two groups and evaluated OS using Kaplan-Meier curves. Patients with a history of aspirin use demonstrated a longer median survival time than patients without a history of aspirin use (92.9 months vs. 87.9 months) (Figure 1). However, this difference was not significant (p = 0.63). Furthermore, even after adjustment for demographic factors, tumor characteristics, and comorbidities, the difference in median OS between aspirin users and non-users remained non-significant (Table 2). Specifically, there were no survival differences by race. Of note, age, grade, and stage were all negative predictors of OS on both univariate and multivariate analyses.

**Table 2:** Univariate and multivariate analysis of potential predictors of overall survival in CRC patients. AJCC: American Joint Committee on Cancer; HTN: Hypertension; HLD: Hyperlipidemia; DM: Diabetes mellitus; CKD: Chronic kidney disease; CRC: Colorectal cancer.

	Univariate		Multivariate	
Characteristic	OR (95% CI)	P-value	OR (95% CI)	P-value
Age	1.030 (1.022-1.039)	0.000	1.031 (1.021-1.040)	0.000
Gender	1.129 (0.921-1.386)	0.234		
Aspirin use	0.950 (0.771-1.170)	0.630	0.830 (0.666-1.034)	0.097
Aspirin use > 1 year	0.837 (0.669-1.048)	0.120		
Chemotherapy	1.241 (1.010-1.525)	0.040	0.882 (0.683-1.140)	0.339
Grade (3&4 compared to 1&2)	2.028 (1.622-2.537)	0.000	1.859 (1.483-2.330)	0.000
AJCC Stage (3&4 compared to 1&2)	2.252 (1.826-2.776)	0.000	2.491 (1.945-3.190)	0.000
Primary site of the tumor (Distal vs Proximal)	0.834 (0.623-1.118)	0.225		
Race				
Whites compared to Asians	1.153 (0.813-1.635)	0.425		
Whites compared to Pacific-Islanders	1.158 (0.741-1.810)	0.519		
HTN	1.027 (0.833-1.266)	0.804		
HLD	0.879 (0.718-1.076)	0.210		
DM	0.909 (0.722-1.146)	0.420		
CKD	1.343 (1.015-1.776)	0.039	1.266 (0.941-1.701)	0.119

## Discussion

Our study showed no association between pre-diagnosis aspirin use and OS in a racially diverse population of CRC patients. This lack of an association persisted even after adjusting for other prognostic factors. This finding is consistent with the current literature. Multiple studies and meta-analyses have failed to show a benefit of pre-diagnosis aspirin use on cancer-specific survival or OS. Two meta-analyses done by Li P et al. and Xiao S et al. showed no evidence of an association between pre-diagnosis aspirin use and improved patients' cancer-specific mortality or OS [9,10]. Another meta-analysis that included 16654 patients with a history of pre-diagnosis aspirin use showed no effect on cancer-specific mortality [11]. Moreover, Gray RT et al., in a retrospective cohort study of 8391 patients showed no reduction in CRC-specific mortality among pre-diagnosis and post-diagnosis low-dose aspirin users [12].

The meta-analysis done by Li P et al. examined the benefit of post-diagnosis aspirin use on survival in patients with CRC and showed an OS benefit, which was true in both colon and rectal cancer, but only for those with positive COX-2 expression or mutated PI3KCA [9]. However, there was no benefit associated with cancer-specific mortality [9]. Furthermore, there are two large ongoing placebo-controlled randomized controlled trials that examine the effect of both aspirin and metformin in stage I-III CRC, and the effect of aspirin in stage III and high-risk stage II colon cancer with PIK3CA mutation, respectively [13,14]. This effect was also shown in a systematic review and meta-analysis that showed the overall effect of aspirin was not significant. However, in cancers with a PI3KCA mutant, aspirin use was associated with a 29% reduction in mortality [15].

Our study has large populations of Asians and Pacific Islanders. The incidence and mortality rates of CRC are low in Asians and Pacific-Islanders in the United States; however, more recent studies suggest that both the incidence and mortality of CRC are rising in Asia [16,17]. There was no association between aspirin use and OS in our cohort even after adjusting for race. A significant percentage (17.7%) of our study population were Pacific Islanders, a group that has not been well-represented in previous studies. This racially patient population enhances the generalizability of the results.

There was a higher incidence of medical comorbidities among patients with a history of aspirin use than patients without aspirin use. However, there were no differences in tumor characteristics, including grade, stage, or location, between the two groups. The absence of an association between aspirin use and negative prognostic factors is consistent with our finding of a lack of an association between aspirin use and OS.

Our study has some limitations. Due to the retrospective nature of the analysis, all data were extracted from patient charts, though efforts were made to limit potential errors. Due to the nature of chart review data, we cannot be certain about patients' compliance with aspirin administration. Moreover, we are not able to determine if patients continued aspirin use after diagnosis. Furthermore, the measured outcome in our study is OS rather than cancer-specific survival, which would be an essential measure to study. On the other hand, our study has a large sample size, as well as a racially diverse population which is unique and adds to the generalizability of our results.

## Conclusions

In this large retrospective study of a racially diverse population, there was no association between pre-diagnosis aspirin use and OS in CRC patients. Our results are consistent with the current evidence from other studies in the literature. However, some studies pointed toward a possible effect of continued aspirin use and post-diagnosis aspirin use on OS and cancer-specific survival in CRC patients. Therefore, physicians should be aware of the potential side effects and weigh the risks and benefits of aspirin use as a potential modulator of CRC prognosis in high-risk patients. Moreover, future studies should be ideally prospective, collect data about aspirin use prior to and after diagnosis, and measure cancer-specific survival as an outcome analysis. 

## References

[1] Siegel RL, Miller KD, Fuchs HE, Jemal A (2021). Cancer Statistics, 2021. CA Cancer J Clin.

[2] Drew DA, Cao Y, Chan AT (2016). Aspirin and colorectal cancer: the promise of precision chemoprevention. Nat Rev Cancer.

[3] Nan H, Hutter CM, Lin Y (2015). Association of aspirin and NSAID use with risk of colorectal cancer according to genetic variants. JAMA.

[4] Burn J, Gerdes AM, Macrae F (2011). Long-term effect of aspirin on cancer risk in carriers of hereditary colorectal cancer: an analysis from the CAPP2 randomised controlled trial. Lancet.

[5] Emilsson L, Holme Ø, Bretthauer M (2017). Systematic review with meta-analysis: the comparative effectiveness of aspirin vs. screening for colorectal cancer prevention. Aliment Pharmacol Ther.

[6] Perisetti A, Goyal H, Tharian B, Inamdar S, Mehta JL (2021). Aspirin for prevention of colorectal cancer in the elderly: friend or foe?. Ann Gastroenterol.

[7] Hamada T, Cao Y, Qian ZR (2017). Aspirin use and colorectal cancer survival according to tumor CD274 (Programmed Cell Death 1 Ligand 1) expression status. J Clin Oncol.

[8] Liao X, Lochhead P, Nishihara R (2012). Aspirin use, tumor PIK3CA mutation, and colorectal-cancer survival. N Engl J Med.

[9] Li P, Wu H, Zhang H (2015). Aspirin use after diagnosis but not prediagnosis improves established colorectal cancer survival: a meta-analysis. Gut.

[10] Xiao S, Xie W, Fan Y, Zhou L (2021). Timing of aspirin use among patients with colorectal cancer in relation to mortality: a systematic review and meta-analysis. JNCI Cancer Spectr.

[11] Wang X, Luo Y, Chen T, Zhang K (2021). Low-dose aspirin use and cancer-specific mortality: a meta-analysis of cohort studies. J Public Health (Oxf).

[12] Gray RT, Coleman HG, Hughes C, Murray LJ, Cardwell CR (2018). Low-dose aspirin use and survival in colorectal cancer: results from a population-based cohort study. BMC Cancer.

[13] Michel P, Boige V, Andre T (2018). Aspirin versus placebo in stage III or high-risk stage II colon cancer with PIK3CA mutation: a French randomised double-blind phase III trial (PRODIGE 50-ASPIK). Dig Liver Dis.

[14] Petrera M, Paleari L, Clavarezza M (2018). The ASAMET trial: a randomized, phase II, double-blind, placebo-controlled, multicenter, 2 × 2 factorial biomarker study of tertiary prevention with low-dose aspirin and metformin in stage I-III colorectal cancer patients. BMC Cancer.

[15] Paleari L, Puntoni M, Clavarezza M, DeCensi M, Cuzick J, DeCensi A (2016). PIK3CA mutation, aspirin use after diagnosis and survival of colorectal cancer. A systematic review and meta-analysis of epidemiological studies. Clin Oncol (R Coll Radiol).

[16] Onyoh EF, Hsu WF, Chang LC, Lee YC, Wu MS, Chiu HM (2019). The rise of colorectal cancer in Asia: epidemiology, screening, and management. Curr Gastroenterol Rep.

[17] Ellis L, Canchola AJ, Spiegel D, Ladabaum U, Haile R, Gomez SL (2018). Racial and ethnic disparities in cancer survival: the contribution of tumor, sociodemographic, institutional, and neighborhood characteristics. J Clin Oncol.

